# Students’ Emotional Well-Being, and Perceived Faculty Incivility and Just Behavior Before and During COVID-19

**DOI:** 10.3389/fpsyg.2022.849489

**Published:** 2022-04-25

**Authors:** Dorit Alt, Yariv Itzkovich, Lior Naamati-Schneider

**Affiliations:** ^1^Kinneret Academic College on the Sea of Galilee, Jordan Valley, Israel; ^2^Tel Hai College, Upper Galilee, Israel; ^3^Hadassah Academic College, Jerusalem, Israel

**Keywords:** emotional well-being, faculty incivility, teacher justice, positive and negative affect, higher education

## Abstract

This research set out to measure the impact of the lockdown condition and social distancing imposed on higher education by the Israeli government during the COVID-19 period and the shift to online learning, on students’ emotional well-being, the way they perceived their teachers’ just behavior, and faculty incivility, compared to pre-pandemic conditions. An additional aim was to explore the set of connections among these factors. The total sample included 396 undergraduate students from three academic colleges. Data were gathered via three questionnaires: Positive/negative affect, Faculty Incivility, and Teacher Justice. Data were analyzed using Partial Least Squares-Structural Equation Modeling (PLS-SEM). The main finding showed that students’ negative emotions were informed by the lockdown condition. This perceived negative affect had an impact on how the participants experienced social interactions with their faculty. Those who exhibited higher levels of negative affect perceived themselves as targets of faculty incivility. The same trajectory was detected with the way students experienced their teachers’ just behavior. Students who held negative emotions, partly because of the COVID-19 restrictions, also viewed their teachers’ behavior toward them as unjust. This study stresses the role of one’s emotional condition in instigating negative interpretations of social interactions. Directions for subsequent research and practical implications for promoting students’ well-being and civil and just communications in the learning environment are discussed.

## Introduction

Students’ well-being has received growing attention in past years (Hascher, 2010; Nishina, 2012; Kuroki, 2013; Young-Jones et al., 2015), and gained more prominence in current studies that deal with the effect of COVID-19 on students’ emotions during this turbulent period (Fernández-Abascal and Martín-Díaz, 2021). The present study adds to the corpus of knowledge by empirically investigating the effect of students’ negative and positive emotions on the way they perceived social interaction with their faculty, before and during the lockdown, imposed by the government on the higher education system in Israel due to COVID-19.

Drawing on the Mood Congruence Theory (Bower, 1981; Bower and Forgas, 2000; Forgas, 2017; Matovic and Forgas, 2018), suggesting that subjects’ interpretations of social behaviors and communication are informed by their moods, this study delves deeper into the emotional mechanism that navigates an individual’s interpretation of social information (Aquino and Bradfield, 2000; Oliver et al., 2010). It mainly asserts that students’ positive or negative affects would influence the way they interpret social interactions with their faculty.

Two constructs of social interaction in the learning environment were measured and discussed in this study. The first illustrates a positive aspect of interaction between teachers and students termed Teacher Justice (TJ), which evaluates the extent to which students perceive their teachers’ behavior toward them as just (Peter and Dalbert, 2010; Alt, 2014). Justice is considered a key component in evaluating the students’ experience of their psychosocial learning environment (Alt and Itzkovich, 2015), therefore its assessment in this study is deemed important. The second, a somewhat underexplored phenomenon, is Faculty Incivility (FI), which pertains to deviant behaviors of faculty members that impede learning and engagement in the classroom (Feldmann, 2001; Miller et al., 2014).

These sets of relationships were examined at two points in time: before and during the COVID-19 period, among college students. The main aim of this process was to compare face-to-face teaching in classrooms with distance learning in times of crisis in terms of students’ perception of social interaction with their teachers (i.e., TJ and FI). Another aim was to reveal the way their emotions (positive/negative) would affect these perceptions in times of instability and uncertainty.

The results of this study could initially reveal the impact of social distancing and the shift to online learning on students’ emotional well-being and the way they interpret social interactions in their learning environment. It may help key stakeholders within higher education institutions, improve preparedness for future situations involving social distancing.

## Literature Review

### Defining Academic Incivility

Incivility was defined by Andersson and Pearson (1999) as a “low-intensity deviant behavior with ambiguous intent to harm the target, in violation of workplace norms for mutual respect” (p. 457). It has initially been investigated mainly in organizational contexts (Miner and Cortina, 2016; Zurbrügg and Miner, 2016), yet, based on the notion that academic institutions share some characteristics with organizations (Marchiondo et al., 2010), incivility researchers have embraced a wider perspective and have expanded their work to include academic institutions-related investigations. Some of those studies focused on the unique characteristics of incivility in academic settings, and broadly defined it as “any action that interferes with a harmonious and cooperative learning atmosphere” (Feldmann, 2001, p. 137; Miller et al., 2014, p. 2). In contrast to the above inclusive perspective, other researchers have suggested a narrower definition, drawing on Andersson and Pearson’s (1999) organizational definition, which addressed only specific instantiations of incivilities, such as insults, disrespectful remarks, and hostile looks or sneers (Caza and Cortina, 2007), which can fit both the workplace and academic institutions alike.

Academic incivility can range from active to passive manifestations (Alt and Itzkovich, 2015, 2016, 2017, 2018). The former includes serious incivilities, such as personal comments or verbal attacks against students; the latter pertains to more subtle incivilities, such as inadequate communications and avoidance. FI has been associated in previous studies with a negative emotional level of adjustment to college life (Alt and Itzkovich, 2016). Faculty uncivil behavior has been associated with perceptions of teachers as unjust (Alt and Itzkovich, 2015), and authoritarian behaviors (Alt and Itzkovich, 2018), and has been correlated strongly with students’ dissatisfaction with their studied program (Marchiondo et al., 2010).

While the above-mentioned studies assessed FI in face-to-face learning environments, few have examined this phenomenon in online settings. For example, in their recent study, Campbell et al. (2020) asserted that the shift to online teaching by an increasing number of faculty also increased the frequency of online incivility instantiations. They defined online academic incivility as “any discourteous verbal or non-verbal behaviors directed toward others, such as instructors, students, or observers that disrupts the online learning environment” (p. 110). It should be noted that although this definition pertains to academic incivility in general perpetrated by instructors, students, or observers alike, the authors mainly discussed online academic incivility activities committed by students and ways by which faculty can navigate these disruptions. The current study suggests exploring an overlooked aspect of this phenomenon, namely students’ perceptions of FI in face-to-face vs. online learning environments and proposes solutions for addressing these detrimental behaviors in online learning during times of crisis.

### Experience of Teacher Justice in the Classroom

According to Peter and Dalbert (2010), the individual perception of TJ behavior could act as a potential personal predictor of the class climate experience. These researchers have investigated the connection between TJ behavior and perceived class climate by gathering data from academic-track students in German secondary schools. Multilevel analysis results have shown that students who evaluated their teachers’ behavior toward them personally as just have also evaluated the class climate more positively. Similarly, Jiang et al. (2018) contended that TJ plays an important role for school-age adolescents’ learning and social outcomes. They examined the relationship between TJ and students’ class identification in 1735 Chinese school-age adolescents. Their findings showed TJ had a positive effect on students’ class identification, suggesting that for adolescents, TJ shapes students’ interpersonal relationships with teachers, and affects their sense of belonging and values in relation to their class.

In the field of higher education, Alt (2014) assessed the connection between the perception of the learning environment, tendency toward academic cheating neutralization, and individual experience of TJ behavior. Her study revealed that students who evaluated their teachers’ behavior toward them personally as just, held a more positive evaluation of the learning environment and were less inclined toward academic cheating neutralization. Additionally, Alt and Itzkovich (2015) showed that undergraduate students who evaluated their teachers’ behavior toward them personally as just, reported on less faculty uncivil occurrences in the classroom; thus, making it likely that the evaluation of TJ behavior is interrelated with both personality and incivility constructs.

Similar to FI studies, TJ has been scarcely explored in online instructional settings, hence, there is relatively little discussion about this issue in professional literature. For example, Syynimaa (2018) investigated teachers’ perspectives about hybrid courses, where students partly participated in both online lessons and classroom lessons. Results showed that teaching in hybrid courses was less favorable among teachers mainly because the online learning environment lacked deep, interactive communication. Online participants utilized merely chat or voice, and some major network problems were detected, which ruled out non-verbal and stable communication. The teachers felt that equality could not be achieved in these circumstances, as the classroom participants received more attention than those enrolled in online settings.

### Students’ Subjective Emotional Well-Being

Students’ well-being as a success indicator has received wide attention in educational research (Hascher, 2010; Nishina, 2012; Kuroki, 2013; Young-Jones et al., 2015; Alessandri et al., 2020) and was found to be related to academic achievements (Nickerson et al., 2011), both as a precursor to, or outcome of, academic success. As an emotional factor, students’ well-being was also considered as an outcome of interpersonal relationships, both positive (King et al., 2012), and adverse (Espinoza et al., 2013).

Well-being includes an affective component and a cognitive-judgmental component. The latter represents an individual’s self-evaluation of satisfaction with their life in general (Diener et al., 2010). The affective component, lying at the core of the present study, is comprised of both positive and negative affects (Snyder and Lopez, 2002). The positive affect encompasses emotional states like joy, which are felt by a person, whereas the negative affect relates to negative emotional states like sadness (Watson et al., 1988).

Generally, negative and positive affects represent a two-dimensional taxonomy of personality, as introduced by Watson and Clark (1984). Positive affect represents a generalized tendency to exhibit high energy and positive emotions such as enthusiasm (Bouckenooghe et al., 2013). Conversely, negative affect refers to the tendency to experience a wide range of negative emotions, such as anger, fear, and sadness across situations (Aquino and Bradfield, 2000; Samnani et al., 2013), even in a stress-neutral environment (Aquino et al., 1999).

Although positive and negative affects represent two adverse predispositions, which influence the perception of ourselves and our surroundings (Chan, 2001), scholars have primarily focused attention on assessing negative emotions, namely the negative affect. For example, some studies focused on the offender’s point of view, showing that high levels of negative affect relate to an increased inclination toward perpetrating interpersonal deviance (Alias et al., 2012). Generally, these perspectives rely on two separate mechanisms. The first is objective and focuses on the rebellious characteristic of those who exhibit a high negative affect. Due to their rebellious behavior, they evoke antagonism and therefore increase their probability of becoming victims of aggressive behavior (Aquino et al., 1999; Spector et al., 2000; Alt and Itzkovich, 2016). The second mechanism is subjective – due to their inclination to interpret social information negatively (Aquino and Bradfield, 2000), and given the frequent negative mood states they experience (Oliver et al., 2010), those who exhibit high levels of negative affect are likely to interpret experiences negatively.

Both negative and positive affects have been utilized in educational psychology – specifically as indicators of well-being (Nickerson et al., 2011). In this respect, it was noted, in congruence with the subjective viewpoint, that individuals tend to capture positive stimuli better when they are in a positive mood, and tend to capture negative stimuli better in a negative mood (Hascher, 2010; Bilderbeck et al., 2017; Li et al., 2017). These premises support the Mood Congruence Theory, suggesting that subjects’ interpretations of social behaviors and communication are informed by their tendency to feel good or bad (Bower, 1981; Bower and Forgas, 2000; Forgas, 2017; Matovic and Forgas, 2018). It should be noted that students’ emotional well-being and the way it may affect their perceptions of FI and TJ in the learning environment were overlooked in previous work.

Recently available studies on COVID-19 and mental health (e.g., anxiety) have pointed to the negative impact of the pandemic on students and the general population (Cao et al., 2020; Roy et al., 2020; Valadez et al., 2020; Wang et al., 2020), while scarcely addressing the effect of the lockdowns on students’ well-being. Nonetheless, the handful of studies found in this context showed a significant decrease in students’ well-being during the confinement imposed by governments due to the spread of COVID-19. For example, Fernández-Abascal and Martín-Díaz (2021) initially evaluated the impact of the COVID-19 pandemic on undergraduate students’ psychological well-being both before and during the confinement, by using the Positive and Negative Affect Scale (PANAS). The analyses revealed a significant decline in the Positive Affect construct between the tests whereas the Negative Affect remained stable. In another longitudinal study (Evans et al., 2021), data were collected from undergraduates of a United Kingdom university at two-time points: pre-pandemic and under “lockdown” conditions. Analyses showed a significant reduction in well-being during lockdowns.

### This Study

**FIGURE 1:**
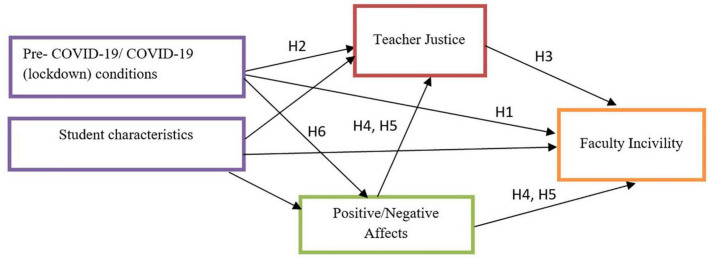
Model 1: The theoretical structure of the proposed framework.

Based on the foundation of the aforementioned studies, the purpose of our research was to measure the impact of the lockdown condition (COVID-19 sample), which imposed a total shift to online learning, on students’ subjective well-being, TJ, and FI perceptions, compared to the pre-pandemic condition (Pre-COVID-19 sample). An additional aim was to explore the set of connections among these factors, in line with the theoretical framework. Accordingly, our main objective was to examine the proposed theoretical Model 1 (Figure 1). The hypotheses set for this study were formulated as:

**H1:** During the COVID-19 period, higher education systems were forced to shift to online learning, leading this period to be seen as a fertile ground for studies that sought to compare the extent of this phenomenon a in face-to-face routine vs. online learning during times of turbulence. Given this innovative line of research, a non-directional hypothesis was devised as: differences would be detected in FI between the Pre-COVID-19 and COVID-19 groups.

**H2:** With the scant research on TJ in online learning environments (Syynimaa, 2018) and specifically during the pandemic, it was hypothesized that differences would be detected between the Pre-COVID-19 and the COVID-19 groups in relation to TJ. Students in face-to-face settings (Pre-COVID-19 group) would report experiencing higher levels of TJ compared with the online participants (COVID-19 group).

**H3:** Drawing on previous studies (Alt and Itzkovich, 2015), the third research question was: how would students’ perception of TJ be connected to their FI perception? It was expected that negative connections would be detected between the variables.

**H4:** Negative Affect would be positively related to FI and negatively to TJ.

**H5:** Positive Affect would be negatively connected to FI and positively to TJ.

**H6:** Higher levels of negative affect would be revealed among the COVID-19 group compared with the Pre-COVID-19 participants.

Finally, background variables, such as reported grade point average, gender, age, and socio-economic status were also addressed in this research to examine how they intersected and impacted the measured variables (Cortina, 2008; Cortina et al., 2013), and to control the differences between the condition groups (Pre-COVID-19 group and COVID-19). Figure 1 demonstrates the theoretical structure of the proposed framework.

## Materials and Methods

### Participants

The total sample included 396 undergraduate students from three public academic colleges located in central Israel, of whom 288 filled out the questionnaires before the breakout of COVID-19, and 108 were sampled during the lockdown in 2020, when the pandemic has resulted in universities being shut all across Israel, leading to a distinctive rise of distance learning, on digital platforms. Data included students’ ethnicity, gender, age, socioeconomic status, and current education achievements. Students’ socioeconomic-status (SES) was assessed by the father’s educational attainment (FEA) and the mother’s educational attainment (MEA), both defined on a six-level scale: 0 = *lack of education*, 1 = *elementary school*, 2 = *high school*, 3 = *BA degree*, 4 = *MA degree*, 5 = *doctoral degree*. Another SES factor was the participants’ report on their economic condition (EC), defined on a six-level scale, from 1 = *extremely difficult* to 6 = *comfortable, no financial worries*. Finally, students’ current education achievements were measured by their self-reported grade point average (GPA). The participants were asked to indicate their GPA on an eight-level scale ranging from 1 = 60–65 to 8 = 96–100. The results are presented in Table 1. Analysis of the differences between the groups (pre-COVID-19 group vs. COVID-19 group) revealed significant differences on the following variables: GPA [*F*_(1_,_390)_ = 6.50, *p* < 0.05], the pre-COVID-19 group reported having higher results than the COVID-19 group; Ethnicity [*F*_(1_,_392)_ = 23.40, *p* < 0.001] the COVID-19 group included more minority students than pre-COVID-19 group; father’s [*F*_(1_,_384)_ = 101.76, *p* < 0.001] and mother’s [*F*_(1_,_391)_ = 111.92, *p* < 0.001] educational attainment, higher results were shown for the COVID-19 group. Other results were found non-significant.

**TABLE 1:** Student characteristics (pre-COVID-19 group and COVID-19 group).

	Pre-COVID-19 group		COVID-19 group	
	*M*	*SD*	*M*	*SD*
Age	26.53	5.29	25.30	8.05
Economic condition (EC)	4.19	1.08	4.04	1.21
Mother educational attainment	2.31	0.98	3.50	1.00
Father educational attainment	2.18	0.98	3.28	1.18
Current education achievements (GPA)	5.64	1.39	5.24	1.43
Gender	80.6% females		86.8% females	
Ethnicity	100% Jewish		93% Jewish 7% Arab	

### Measurements

#### Positive/Negative Affect

The positive and negative affect scale (PANAS), developed by Watson et al. (1988), includes 20 items. The scale consists of 10 words describing negative emotions (e.g., sad, upset, guilty, etc.) and 10 words describing positive emotions (e.g., interested, enthusiastic, proud, etc.). Respondents were asked to indicate on a Likert-type score, ranging from 1 = *not at all* to 6 = *very much*, to what extent they had experienced the 20 emotions in the past 6 months. All items were subjected to principal component analysis followed by a varimax rotation with eigenvalue >1.00 as a criterion for determining the number of factors. The analysis for the pre-COVID-19 sample resulted in two factors, which accounted together for 57.19% of the variance. Similarly, the results for the COVID-19 sample yielded a two-factor structure, which accounted for 55.38% of the variance. Computed internal consistencies (Cronbach’s alpha) for each factor in each sample ranged from 0.904 < α < 0.921 indicating sufficient reliability results within the factors.

#### The Perceived Faculty Incivility Scale

This scale was designed by Alt and Itzkovich (2015, 2016, 2017, 2018) to measure the frequency of faculty incivility occurrences. It includes two factors: Factor I contained 13 items representing Active Incivility, for example, “The teacher yells at you as a response to misunderstanding.” Factor II contained eight items pertaining to Passive Incivility, for example, “The teacher ignores students’ questions during lectures.” Each item was given a Likert-type score ranging from 1 = *almost never* to 5 = *nearly always*. Several items were slightly adapted when used to assess FI during the COVID-19. For example, the latter item was phrased “The teacher ignores students’ questions during online (zoom) lectures.” Data gathered from each sample were subjected to principal component analysis followed by Varimax rotation with eigenvalue >1.00 as a criterion for determining the number of factors. The analysis for the pre-COVID-19 sample resulted in two factors, which accounted together for 42.22% of the variance. In a similar vein, the results for the COVID-19 sample yielded a two-factor structure, which accounted for 52.06 of the variance. Computed internal consistencies (Cronbach’s alpha) for each factor in each sample ranged from 0.744 < α < 0.924 indicating sufficient reliability results within the factors. One Passive Incivility item was deleted due to a low item loading result from both samples.

#### The Teachers’ Justice Scale

This scale (Dalbert and Stöber, 2002) is used to measure students’ perception of their teachers’ just behavior toward them. This six-point Likert-style format scale (from 1 = *I totally disagree* to 6 = *I totally agree*) includes items such as, “My teachers generally treat me fairly.” The original scale includes 10 items, however, three items that are less related to college study settings (e.g., “My grades are often based on my behavior rather than on my achievements”), have been excluded from the original 10-item questionnaire (α _*pre–COVID–*19_ _*group*_ = 0.94; α _*COVID–*19_ _*group*_ = 0.95).

Table 2 displays the descriptive statistics of the research constructs and indicators. Following the general guidelines for skewness and kurtosis (suggesting that if the number is greater than +1 or lower than −1, then the distribution is skewed, flat, or peaked), it can be learned that the active FI distribution can be considered non-normal. Based on previous studies (Alt and Itzkovich, 2015, 2016, 2017, 2018) that might have been expected. However, it should be noted that in situations where it is difficult to meet the strict requirements of more traditional multivariate techniques, such as normal data distribution, Partial Least Squares-Structural Equation Modeling (PLS-SEM) should be considered as a preferred method. PLS-SEM has greater flexibility in this respect compared with covariance-based SEM (CB-SEM) when generally making no assumption about the data distribution.

**TABLE 2:** Descriptive statistics of the research constructs.

Construct	*Mean*	*SD*	Skewness	Kurtosis
Passive FI	1.91	0.55	0.849	1.32
Active FI	1.41	0.51	2.34	8.43
TJ	4.93	0.97	–1.20	1.82
Positive affect	3.41	0.84	0.112	0.612
Negative affect	2.03	0.81	0.989	0.952

### Procedure

Participants were recruited by placing internet ads in students’ forums of three public academic colleges, located in central Israel, inviting undergraduate students to participate in the research. Prior to obtaining participants’ consent, it was explained that the questionnaires were anonymous and that it was acceptable should they choose to submit a partially completed questionnaire. Finally, participants were assured that no specific identifying information would be processed. The study was preauthorized by the college’s Ethics Committee.

### Data Analysis

Data were analyzed using analyses of variance to compare variances across the means of different groups and PLS-SEM. Whereas CB-SEM is primarily used for confirmation of a founded theory, PLS is a prediction-oriented approach to SEM, primarily used for exploratory research, therefore, advised to be employed if the primary objective of applying structural equation modeling is the prediction of target constructs (Hair et al., 2017). SmartPLS 3 software was used.

## Results

In H1 it was postulated that differences would be detected in FI (passive and active) between the pre-COVID-19 and COVID-19 groups. A multivariate analysis of variance (MANOVA) was found significant [*F*_(2_,_393)_ = 5.0; *p* < 0.001; η^2^ = 0.03]. Concerning the passive FI variable, a univariate analysis of variance (ANOVA) was found non-significant [*F*_(1_,_394)_ = 0.6; *p* > 0.05; η^2^ = 0.00]. As for the active FI variable, the ANOVA was found significant [*F*_(1_,_394)_ = 8.22; *p* < 0.01; η^2^ = 0.02]. As can be seen in Table 2, a higher mean result was reported by the pre-COVID-19 group compared with the COVID-19 group. *H1* was partially corroborated.

In H2 it was conjectured that the pre-COVID-19 group would report experiencing higher levels of TJ compared with the COVID-19 group. A univariate analysis of variance (one-way ANOVA) was found significant [*F*_(1_,_393)_ = 11.15; *p* < 0.01; η^2^ = 0.03]. However, in contrast to our speculation, as can be learned from Table 2, the COVID-19 group (online learning) reported higher levels of TJ compared with the pre-COVID-19 group (face-to-face learning). *H2* was not corroborated.

**FIGURE 2:**
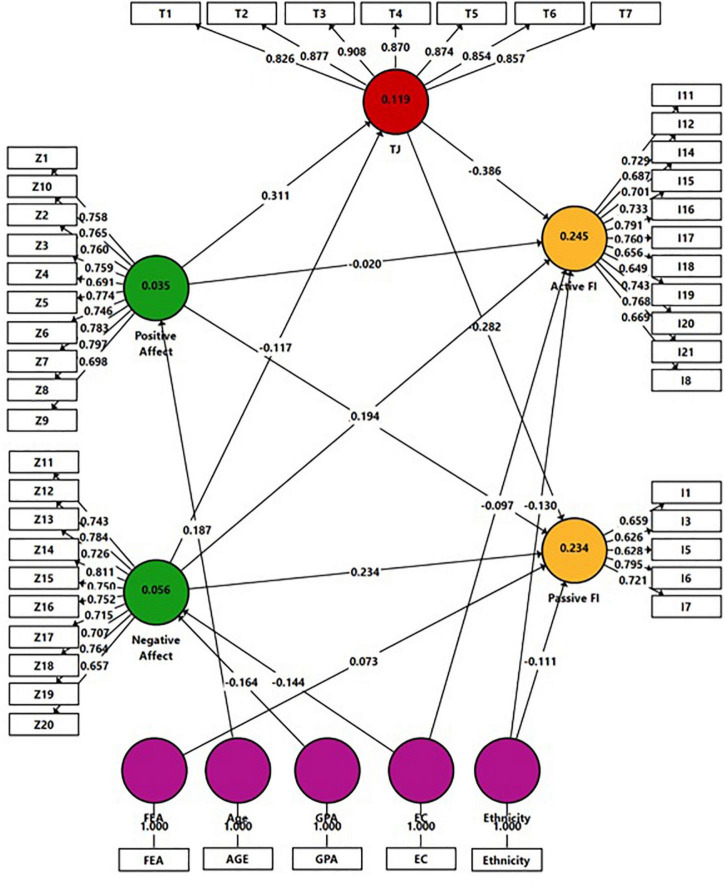
Model 2: Analysis results of the examination of *H3–H5* by SmartPLS.

To assess H3, H4, and H5, Model 2 (Figure 2) was constructed. The model includes the following latent constructs: the two PANAS independent factors (positive/negative affects), TJ, FI, and background variables. This model was designed in two steps. In Step 1, all background variables were entered into the model. The results of this analysis are shown in Table 3. In Step 2, only variables bearing significant links to the constructs presented in Model 2 were entered into the model (see path coefficients highlighted in Table 3).

**TABLE 3:** Significance analysis of the direct and effects of background variables on research constructs.

Path	Direct effect	*t-*value	*p-*value
Age -> Active FI	–0.015	0.325	0.745
Age -> Negative affect	–0.113	1.713	0.087
Age -> Passive FI	–0.010	0.205	0.838
**Age -> Positive affect**	**0.201**	**4.196**	**0.000**
Age -> TJ	0.045	1.034	0.302
**EC -> Active FI**	−**0.101**	**2.340**	**0.020**
**EC -> Negative affect**	−**0.149**	**2.923**	**0.004**
EC -> Passive FI	–0.065	1.412	0.159
EC -> Positive affect	0.091	1.748	0.081
EC -> TJ	0.032	0.657	0.512
**Ethnicity (minority) -> Active FI**	−**0.126**	**3.176**	**0.002**
Ethnicity (minority) -> Negative affect	0.162	1.947	0.052
**Ethnicity (minority) -> Passive FI**	−**0.119**	**2.530**	**0.012**
Ethnicity (minority) -> Positive affect	0.097	1.483	0.139
Ethnicity (minority) -> TJ	–0.013	0.329	0.742
FEA -> Active FI	0.093	1.587	0.113
FEA -> Negative affect	0.107	1.727	0.085
**FEA -> Passive FI**	**0.112**	**2.085**	**0.038**
FEA -> Positive affect	0.099	1.751	0.081
FEA -> TJ	0.079	1.295	0.196
GPA -> Active FI	0.054	1.174	0.241
**GPA -> Negative affect**	−**0.145**	**3.022**	**0.003**
GPA -> Passive FI	–0.086	1.777	0.076
GPA -> Positive affect	–0.023	0.430	0.668
GPA -> TJ	–0.059	1.124	0.262
Gender -> Active FI	–0.089	1.757	0.080
Gender -> Negative affect	–0.007	0.123	0.902
Gender -> Passive FI	–0.067	1.400	0.162
Gender -> Positive affect	0.060	1.190	0.234
Gender -> TJ	–0.015	0.309	0.758
MEA -> Active FI	–0.081	1.415	0.158
MEA -> Negative affect	0.012	0.200	0.842
MEA -> Passive FI	–0.060	0.934	0.351
MEA -> Positive affect	–0.056	0.971	0.332
MEA -> TJ	0.081	1.273	0.203

*Values in bold indicate statistically significant results. Mother’s educational attainment (MEA), father’s educational attainment (FEA).*

Four FI items were omitted from the model due to low loading results (<0.60). Relationships between the constructs are displayed as arrows, in line with the theoretical model (Figure 1). In PLS-SEM, single-headed arrows, as shown between the constructs, are considered predictive relationships, and with a robust theoretical framework, can be construed as causal relationships. A path weighting scheme and a mean value replacement for missing values were used. To test the direct and indirect effects, the bootstrap routine was employed. The results are shown in Model 2 and Table 4.

**TABLE 4:** Significance analysis of the direct and indirect effects.

Path	Direct effect	*t-*value	*p-*value	Indirect effect	*t*-value	*p*-value
Age -> Positive affect	0.187	3.645	0.000			
EC -> Active FI	–0.097	1.988	0.047			
EC -> Negative affect	–0.144	2.751	0.006			
Ethnicity -> Active FI	–0.13	3.244	0.001			
Ethnicity -> Passive FI	–0.111	2.489	0.013			
FEA -> Passive FI	0.073	1.380	0.168			
GPA -> Negative affect	–0.164	3.139	0.002			
Negative affect -> Active FI	0.194	4.136	0.000			
Negative affect -> Passive FI	0.234	5.205	0.000			
Negative affect -> TJ	–0.117	2.125	0.034			
Positive affect -> Active FI	–0.02	0.368	0.713			
Positive affect -> Passive FI	–0.168	3.481	0.001			
Positive affect -> TJ	0.311	7.217	0.000			
TJ -> Active FI	–0.386	6.437	0.000			
TJ -> Passive FI	–0.282	5.043	0.000			
Negative affect -> TJ -> Active FI				0.045	1.885	0.060
Negative affect -> TJ -> Passive FI				0.033	1.888	0.060
Positive affect -> TJ -> Active FI				–0.120	4.826	0.000
Positive affect -> TJ -> Passive FI				–0.088	4.001	0.000

According to *H3*, negative connections were expected between TJ and FI. As shown in Table 4, TJ was found negatively connected to both active and passive FI. *H3* was approved. In *H4* it was hypothesized that Negative Affect would be positively related to FI, and negatively to TJ. Path coefficient results have confirmed this hypothesis. Both active and passive FI were increased by the Negative Affect construct, whereas TJ was negatively informed by this construct. As for the Positive Affect impact on FI, merely passive FI was negatively informed by it, while a non-significant result was shown for active FI. In relation to TJ, as postulated, a positive connection was found between this variable and Positive Affect, hence *H5* was partially confirmed.

Significant indirect effect results pointed to the mediating role of TJ in linking Positive Affect to FI constructs. The latter were negatively impacted by Positive Affect *via* TJ. It should be noted that non-significant coefficient results were found between the Negative Affect and FI mediated by TJ.

In *H6* it was postulated that higher levels of Negative Affect would be revealed in the COVID-19 group compared with the pre-COVID-19 participants. A one-way ANOVA [*F*_(1_,_387)_ = 23.57; *p* < 0.001; η^2^ = 0.06] showed that the COVID-19 group (online learning) reported higher levels of Negative Affect compared with the pre-COVID-19 group (face-to-face learning), as shown in Table 2. *H6* was confirmed.

Regarding background variables, Age was positively connected to Positive Affect, and negatively to Negative Affect. Meaning that younger participants were more inclined toward exhibiting negative emotions. Higher levels of EC and GPA decreased the levels of the Negative Affect variable.

### Model Evaluation

Collinearity was examined by the Variance Inflation Factor (VIF) values. The results showed that the VIF values of all combinations of endogenous and exogenous constructs were below the threshold of 5 (Hair et al., 2017) ranging from 1.00 to 1.40. The coefficient of determination (*R*^2^) value was examined, when *R*^2^ values of 0.75, 0.50, or 0.25 for endogenous latent variables can be described, respectively, as substantial, moderate, or weak (Hair et al., 2017). As can be learned from Model 2, the highest *R*^2^ result was indicated for Active FI (0.25), and the lowest for Positive Affect (0.04). In addition, *f*^2^ effect size was measured, when values of 0.02, 0.15, and 0.35, respectively, represent small, medium, and large effects. The highest *f*^2^ effect size result was shown for TJ -> Active FI (0.174), and the lowest for Positive Affect -> Active FI (0.00). Finally, the predictive relevance (*Q*^2^) of the path model was calculated, when values higher than 0 suggest that the model has predictive relevance for a certain endogenous construct (Hair et al., 2017). The *Q*^2^ value for Active FI was 0.120; Passive FI: 0.104; TJ: 0.089; Negative Affect: 0.031; and Positive Affect: 0.019.

## Discussion

This study was designed to measure students’ perceived emotional well-being, TJ, and FI at two points in time: Pre-COVID-19 and during COVID-19. Another aim was to assess the impact of the lockdown imposed by the government on higher education institutions due to the pandemic, on students’ emotional well-being, TJ, and FI perceptions, and to identify relationships among these factors.

According to the analyses, the “lockdown” condition only affected students’ active FI perceptions. A higher mean result was reported by the pre-COVID-19 group compared with the COVID-19 group. Meaning that the students experienced more FI behaviors in face-to-face settings than in online courses. Previous studies (Campbell et al., 2020) mainly explored academic incivility perpetrated by students in online learning settings. This study adds to past work by showing that from the students’ perspective, online learning strategies diminish active FI instantiations. This can be explained by several studies published during the COVID-19 (Kalman et al., 2020; Danchikov et al., 2021) underscoring the overall poor interaction between teachers and students and poor connectivity due to multiple simultaneous users in online courses. According to others (Ba̧czek et al., 2021; Zboun and Farrah, 2021), students reported that they were less active and experienced less interaction with the facilitators during online classes compared to face-to-face courses. Others reported that students kept online classes with muted microphones and went ahead doing what they want (Pallathadka, 2021). It thus might be inferred that the poor interactions between learners and facilitators compared to face-to-face interactions offered fewer opportunities also for negative communications.

Moreover, the “lockdown” condition did not impede the way students perceived faculty behavior toward them as just. Contrary to our expectation, the COVID-19 condition group reported higher levels of this variable compared with the Pre-COVID 19 group. Our postulation was based on the scant research on TJ in online vs. frontal learning environments, which evaluated the teachers’ point of view. Our study adds to previous work by suggesting that students in online learning, during times of uncertainty, may experience TJ in a more favorable way. This can be due to constructivist learning practices implemented in online courses during the lockdown (Alt and Naamati-Schneider, 2021), enabling teaching practices to adapt to the students’ needs.

Nonetheless, the most important finding showed that students’ negative emotions were informed by the lockdown condition. Differently stated, the group of students sampled during COVID-19 exhibited higher levels of negative emotions than the Pre-COVID-19 participants. This perceived subjective negative affect had an impact on how the participants experienced social interactions with their faculty. Those who exhibited higher levels of negative emotions perceived themselves as targets of FI, similar to high negative affect exhibitors who perceive themselves as targets of abusive supervision in the workplace (Wang et al., 2015).

Two distinct mechanisms can explain these findings. The first focuses on the rebellious characteristic of those who exhibit high negative affect. Due to their rebellious behavior, high negative affect exhibitors arouse resentment, and therefore increase the likelihood of becoming victims of aggressive behavior including, but not limited to, incivility (Spector et al., 2000). The second mechanism is subjective. Due to their predisposition to interpret even neutral social information negatively (Aquino and Bradfield, 2000), and given the frequent negative mood states high negative affect exhibitors experience (Oliver et al., 2010), they are likely to interpret experiences negatively, and therefore perceive neutral gestures as manifestations of FI. In contrast, those who exhibit high levels of positive emotions are expected to feel less resentful and therefore to experience fewer manifestations of FI, as also corroborated by the empirical model assessed in the current research.

The same trajectory was detected with TJ experiences. In line with our findings, students who held negative emotions, partly because of the COVID-19 restrictions, also viewed their teachers’ behavior toward them as unjust. However, when not mediated with emotions, as indicated earlier, TJ was positively informed by the lockdown group of students who reported higher levels of just behavior practiced by their teachers. Therefore, this study underscores the role of emotional conditions in instigating negative interpretations of social interactions.

Additionally, we found that TJ partially mediates the relationship between students’ subjective emotions and FI. However, the mediating effect was only significant in associating positive affect exhibitors with FI. A plausible explanation may be that while the incivility perception of high negative affect exhibitors is rigid, the perception of those demonstrating high levels of positive affect is flexible and their incivility perception depends on the level to which they perceive their teacher as just (Afolabi, 2017). It could also imply that academic institutes should strengthen positive emotional competencies among students as part of an overall plan to mitigate the way they interpret social interactions, specifically during times of instability.

Taken together, our findings possibly support the well-established theory of mood congruence (Bower, 1981), which associates moods with subjects’ interpretations of social behaviors (Bower and Forgas, 2000). Thus, our negative/positive feelings might impact our impressions of, and communication with, other people and the way we interpret social interactions. Following this line of thought, it might be inferred that those who exhibit a negative mood are more receptive to negative stimuli, and alternatively, individuals who exhibit a positive mood tend to capture positive stimuli better.

Regarding student characteristics, the results primarily showed that age, grade point average, and economic conditions were negatively connected to negative emotions. It may be inferred that those who reported having lower academic achievements and inferior economic conditions tended to feel deprived. This can be explained by the relative deprivation theory (Walker and Smith, 2002), suggesting that individuals constantly equate their resources (status, salary, relationship, etc.) to those possessed by others in their surroundings. Mishra and Carleton (2015) reported that perceived deprivation is associated with poor mental health. Among other indicators, the authors lent credence to the relationship between perceptions of deprivation and feelings of nervousness and despondency, which are the ingredients of negative affect. In turn, these emotions can lead to distress and attrition (Mestan, 2016). Based on this premise, it is plausible that lower GPA levels and economic conditions might evoke an inclination toward a negative affect exhibition which consequently can shape the way students view social situations (Feldman and Turnley, 2004). Lastly, younger participants reported higher levels of negative emotions. These findings support the previous studies’ account of positivity effects in older adults (Knight et al., 2016).

### Limitations and Directions for Future Research

The present work features several limitations and further directions for future research that warrant mentioning. First, it should be noted that the cross-sectional nature of the data can prevent definitive statements about causality. Indeed, some relationships in the model are likely reciprocal. For example, our analysis implies that high levels of negative affect could lead to increased perceived FI. However, although this interpretation was supported by previous studies suggesting that negative affect is more likely to predict incivility rather than being an outcome of it (Oliver et al., 2010), others showed that negative affect could be seen as an outcome of adverse social interaction, and not only as its predictor (Lightsey et al., 2012). Although our findings support the former, given the alternative explanations, they should be interpreted with caution.

A second concern is related to the explained variance of FI ranging between 23% and 25%. This may indicate that the model tested here can be expanded in future research by using additional variables related to personality measures, such as self-esteem, positive thinking (Lightsey et al., 2012), or other variables such as power relations between students and teachers (Alt and Itzkovich, 2018), and the way teachers construct the learning environment (Itzkovich et al., 2020). The current study also used self-report measures. Different approaches to survey measurement, as well as experimental and qualitative techniques, should be employed by future researchers in order to overcome common method bias.

Future studies should also inspect the relevance of the victimization theory on how students interpret social situations (Aquino and Thau, 2009; Jiang et al., 2019), similarly to studies on workplace incivility, which is considered a subset of victimization (Alt and Itzkovich, 2016). Victimization broadly focuses on aggressiveness from the viewpoint of its victims, thus placing an emphasis on different precursors that potentially increase the probability of being victimized in social interaction. Among different victim-centered outcomes, negative emotions were considered a potential predictor of victimization (Aquino and Thau, 2009) as well as incivility (Alt and Itzkovich, 2015, 2016). Therefore, future studies might find the victimization theory a useful framework for further exploring civil communications in the classroom (Henle and Gross, 2014).

### Conclusion and Practical Implications

This study elaborates on previous studies by assessing emotional well-being as a precursor of FI and TJ. Despite mounting reports on students’ well-being as an outcome (Bailey and Phillips, 2016; Bore et al., 2016), minimal attention has been devoted to assessing this variable as a precursor. Nonetheless, those who have measured well-being as a precursor (Mestan, 2016) overlooked its potential relation to faculty adverse or just behavior in higher education, and none of them inspected these phenomena during the COVID-19 period.

The implications of these findings on FI go beyond objectivity. The subjective nature of FI as viewed by students, who exhibit different emotions due to an external unstable condition, is underscored, rather than the objective aspect of uncivil instances. This study notes that subjective perceptions, which are highly dependent on well-being indicators, might influence the way we judge events.

Practically, this study’s main results raise the necessity to design strategies that might enhance students’ emotional well-being in general, and specifically in times of crisis, as it might affect the way students interpret interpersonal relationships (Nickerson et al., 2011). Academic institutes should develop and implement programs to strengthen the well-being of students and at the same time present practices and strategies for faculty that could help in building positive communication, such as active listening, and communication of clear and consistent expectations. Our findings show that such activities are ever more required in times of uncertainty. For example, practices suggested to support students’ well-being in times of physical distancing (Crawford, 2020; Dodd et al., 2021) include knowing students’ needs, being aware of challenges incurred by the unstable condition, facilitating student connections, providing students with opportunities to ask questions and receive timely answers, enhancing university’s services, making students feel that their teachers care for them, offering financial and academic support, and ensuring students are aware of university support services available to them during the crisis. These measures may increase students’ positive emotions, which in turn, might enhance positive interpretations of social behaviors and communication in the learning environment; thereby nurturing students’ learning and performances, satisfaction, and retention (Lasiter et al., 2012).

## Data Availability Statement

The datasets presented in this study can be found in online repositories. The names of the repository/repositories and accession number(s) can be found in the article/supplementary material.

## Ethics Statement

The studies involving human participants were reviewed and approved by the Kinneret College. The patients/participants provided their written informed consent to participate in this study.

## Author Contributions

DA: conceptualization, data curation, and writing – original draft preparation and reviewing and editing. YI: conceptualization, data curation, original draft preparation, and writing – reviewing and editing. LN-S: data curation and writing – original draft preparation and reviewing and editing. All authors contributed to the article and approved the submitted version.

## Conflict of Interest

The authors declare that the research was conducted in the absence of any commercial or financial relationships that could be construed as a potential conflict of interest.

## Publisher’s Note

All claims expressed in this article are solely those of the authors and do not necessarily represent those of their affiliated organizations, or those of the publisher, the editors and the reviewers. Any product that may be evaluated in this article, or claim that may be made by its manufacturer, is not guaranteed or endorsed by the publisher.
